# Breast edema, from diagnosis to treatment: state of the art

**DOI:** 10.1186/s40945-021-00103-4

**Published:** 2021-03-29

**Authors:** Hanne Verbelen, Wiebren Tjalma, Dorien Dombrecht, Nick Gebruers

**Affiliations:** 1 Department of Rehabilitation Sciences and Physiotherapy (REVAKI-MOVANT), Faculty of Medicine and Health Sciences, University of Antwerp, Universiteitsplein 1, 2610 Antwerp, Belgium; 2 Faculty of Medicine and Health Sciences, University of Antwerp, Universiteitsplein 1, 2610 Antwerp, Belgium; 3 Multidisciplinary Breast Clinic Antwerp, Antwerp University Hospital (UZA), Wilrijkstraat 10, 2650 Edegem, Belgium; 4 Oedema Clinic, Antwerp University Hospital and University of Antwerp, Drie Eikenstraat 655, 2650 Edegem, Belgium

**Keywords:** Breast neoplasms, Breast edema, Diagnosis, Management

## Abstract

**Introduction:**

Breast edema can arise from different etiologies; however, it is mostly seen after breast conserving surgery and/or radiotherapy. Combining breast conserving surgery and radiotherapy can cause damage to the lymphatic system and reactions to surrounding tissues, which can lead to breast edema; hereby, the breast size can increase by more than one cup size. Swelling of the breast is not the only criterion associated with breast edema. Other common criteria found in literature are peau d’orange, heaviness of the breast, skin thickening, breast pain, redness of the skin, hyperpigmented skin pores and a positive pitting sign. Despite the benefits of breast conserving surgery, breast edema can be uncomfortable, and can negatively influence quality of life in suffering patients. In contrast to lymphedema of the arm, which is well known in clinical practice and in research, breast edema is often underestimated and far less explored in literature. Currently, many aspects still need to be reviewed.

**Purpose and importance to practice:**

This masterclass aims at providing the state of the art of breast edema for all health care workers and researchers involved in the treatment and monitoring of breast cancer patients. It includes current and future perspectives on its diagnosis, longitudinal course and treatment. Furthermore, recommendations for clinical practice and future research are discussed.

**Clinical implications:**

It is recommended to closely monitor those patients in whom breast edema symptoms do not decline within 6 months after termination of radiotherapy and provide them with the appropriate therapy. Since evidence concerning the treatment of breast edema is currently lacking, we recommend the complex decongestive therapy (CDT) to the utmost extent, by analogy with the lymphedema treatment of the extremities. This treatment involves skin care, exercise therapy and compression. Additionally, all patients should be informed about the normal course of breast edema development.

**Future research priorities:**

A consensus should be reached among clinicians and researchers concerning the definition, assessment methods and best treatment of breast edema. Furthermore, high quality studies are necessary to prove the effectiveness of the CDT for breast edema.

**Supplementary Information:**

The online version contains supplementary material available at 10.1186/s40945-021-00103-4.

## Background

**Fig. 1:**
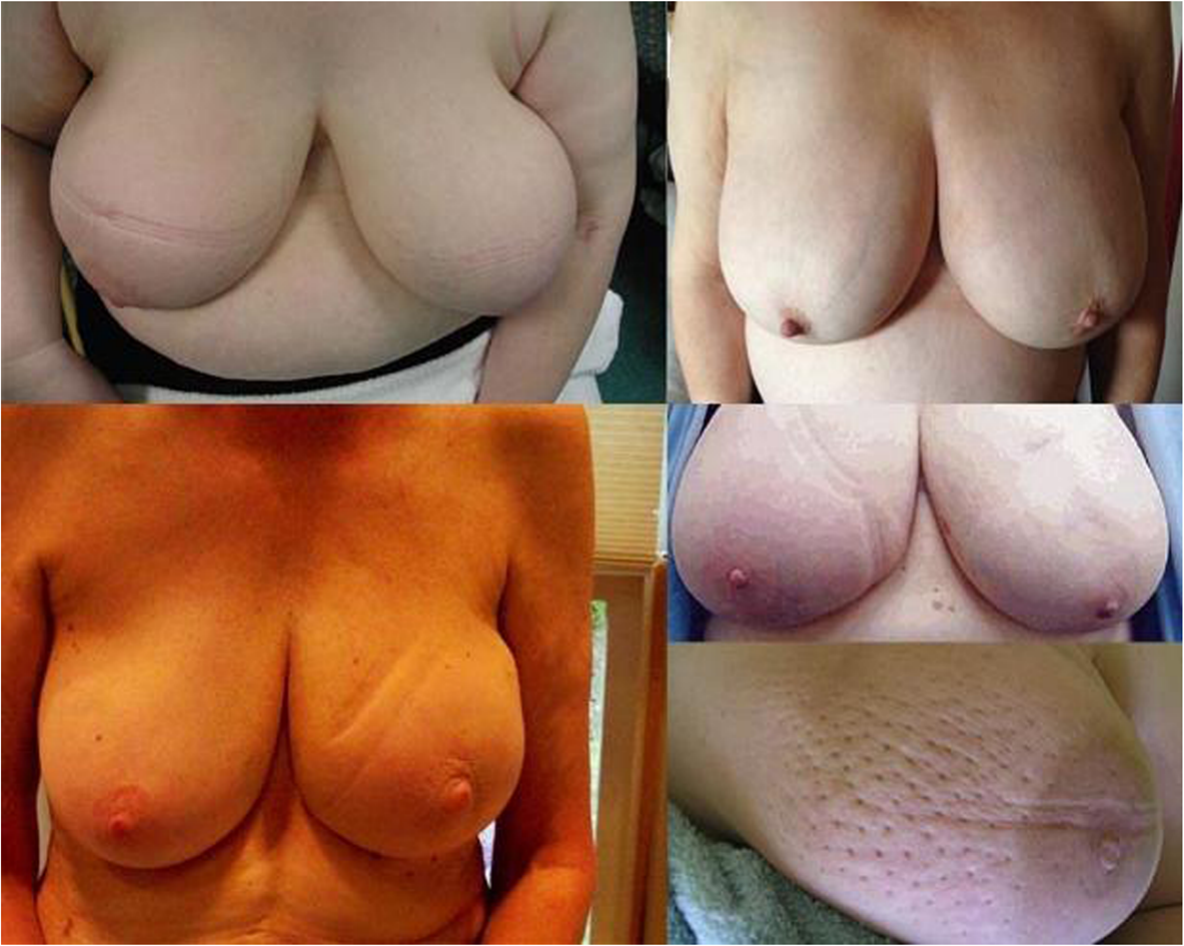
Examples of women suffering from breast edema. The increased volume (including the pitting) is seen on all pictures. In the lower left picture an irregular shape of the breast is seen and the lower right is an example of peau d’orange

Breast cancer is the most common malignancy in women in the Western World [1]. Over the years, breast cancer surgery has evolved to more conservative procedures, as for example breast-conserving surgery (BCS). In most cases this procedure involves radiotherapy, in addition to the local excision. BCS followed by radiotherapy is a safe and effective procedure to treat patients with early stage breast cancer [2]. However, some patients will be troubled by breast edema in the operated and irradiated breast. Breast edema is far less explored in literature compared to lymphedema of the arm. Although, it is gaining relevance due to the increase in patients receiving BCS together with adjuvant radiotherapy. Both aspects of this treatment can cause breast edema. The surgery itself can cause damage to the lymphatic system, which can lead to a compromised transport capacity not only in the arm, but also in the breast. However, the main contributing factor is radiotherapy, which causes various tissue reactions, including edema. Furthermore, venous and lymphatic obstruction could take part in de development of breast edema [3]. In breast edema patients, the breast size can increase by more than one cup size [4]. However, swelling is not the only criterion that is associated with breast edema. Besides an increased volume of the breast [5–10], other common criteria found in literature are peau d’orange [4–6, 8–10], heaviness of the breast [5, 8, 9], redness of the skin [5, 6, 10], breast pain [4–6, 9, 10], skin thickening [6, 11], hyperpigmented skin pores [10] and a positive pitting sign [6] (see Fig. 1). Nevertheless, many studies do not describe a definition for breast edema, making it a difficult topic to study. Clinically, a difference between breast edema and lymphedema of the extremities can be observed. Breast edema is characterized by skin changes, hardness of the breast and pain, but can also be present without visible swelling, whilst the main property of lymphedema of the extremities is swelling. Irradiation causes hardening of the fat tissue. Since a female breast contains lots of adipose tissue, it is likely to undergo those changes post-radiation [12].

Besides surgery and radiotherapy for breast cancer, breast edema can have other etiologies, which are however less common: inflammatory breast carcinoma, metastasis, breast lymphoma, mastitis, fat necrosis, trauma, congestive heart failure etcetera [3]. Therefore, a patient’s clinical history and examination is very important to set an accurate diagnosis and to give appropriate advice or treatment. In contradiction to the natural course of breast edema provoked by BCS and radiotherapy; breast edema from other etiologies often has a chronic stage [3].

Delay et al. classified breast edema into different stages [9]. Stage 1 is characterized by thickening of the skin, while the breast volume remains unchanged. In stage 2, breast edema presents as a visible edema which can lead to asymmetry between both breasts. In patients with severe breast edema, the volume of the operated and irradiated breast can sometimes increase up to 300 ml. Stage 2 is further characterized by dilated skin pores, which is called peau d’orange, heaviness, pain and pitting edema on the affected breast. Stage 3 of breast edema is similar to stage 2, but in this stage the pain is more extensive [9]. Wratten et al. describes 2 components of breast edema. Firstly, generalized enlargement or swelling of the breast tissue itself may occur, which is referred to as parenchymal breast edema. Secondly, there may be evidence of edematous changes in the epidermis and dermis, which is referred to as cutaneous breast edema. Although cutaneous breast edema may occur by itself, in many instances, there will be a combination of both components [11]. Besides the absence of a clear definition for breast edema, there is no standardized method to assess breast edema neither. The most common method found in literature is the physical examination [4, 6, 7, 13–29]. Other assessment methods are mammography [16, 30], ultrasound [6, 11, 16], MRI [31], the tissue dielectric constant (TDC) technique using the MoistureMeterD [32] or questionnaires [5, 23, 26, 33, 34]. Based on a systematic review of the literature, the overall incidence of breast edema following BCS and radiotherapy ranges between 0 and 90.4% [35]. This range includes all kinds of assessment methods and definitions of breast edema and is therefore very broad. Furthermore, evidence on the treatment of breast edema is lacking as well. Therefore, in this paper we provide recommendations based on the current knowledge of lymphedema treatment of the limbs, namely the complex decongestive therapy (CDT). This masterclass is established based on systematic review of the current scientific literature using Pubmed, Embase, Web of Science and Cochrane clinical trials and original prospective research, in the context of a doctoral dissertation. In addition, it is based on clinical experience. It aims at providing the state of the art of breast edema for all health care workers and researchers involved in the treatment and monitoring of breast cancer patients. It includes current and future perspectives on its diagnosis, longitudinal course and treatment. It involves recommendations for clinical practice and for future research.

## Management of breast edema

### Diagnosis

In 2014 a rigorous systematic review was published on the topic of breast edema concluding that a standardized protocol to assess breast edema as well as a clear definition for diagnosis was lacking [35]. A physical examination is the most commonly used method found in literature to assess breast edema in which symptoms of breast edema are evaluated by means of inspection, palpation and anamnesis [4, 6, 15, 16, 19–23, 25, 26, 29]. Additionally, clinical pictures of the breasts could be taken in order to assess the evolution more accurately [7, 17, 28]. Furthermore, several imaging techniques are described in literature, for instance high-frequency ultrasound (HFUS). Clinical signs of breast edema on HFUS are thickening of the skin over 2 mm with increased echogenicity, disturbance or poor visibility of the deeper echogenic line and interstitial fluid accumulation [6, 11, 36]. An MRI allows to detect fluid-containing formations such as parenchymal and cutaneous breast edema, which are visible as white areas [31]. On mammography, parenchymal breast edema is seen as trabecular thickening and cutaneous breast edema as skin thickening [30]. Another technique that could provide information on breast edema is TDC, measured with the MoistureMeterD. This device can measure local tissue water to the depth of 2.5 mm. A TDC ratio between the affected and healthy breast, equal to or greater than 1.40, is seen as breast edema [37]. As a result of the different definitions and assessment methods used; breast edema incidence range is very broad [35]. With this conclusion in mind, the Breast Edema Questionnaire (BrEQ) was developed [34]. This Dutch questionnaire is the first, with evidence of validity and reliability, for assessing breast edema in breast cancer patients. Furthermore, the synthesis of symptoms listed in the BrEQ, can be a catalyst to develop a standard definition for breast edema. In the first part of the questionnaire, symptoms of breast edema are scored on a scale from 0 to 10: pain, heaviness, swelling, tensed skin, redness, pitting sign, enlarged skin pores and hardness. Taking into account the International Classification of Functioning, Disability and Health (ICF), several activity limitations and participation restrictions are scored from 0 to 10 in part 2. Clinimetric properties of the BrEQ were tested in a group of breast cancer patients who underwent BCS and radiotherapy. An overview of these clinimetric properties is presented in Table 1. It shows that the BrEQ is a reliable and valid Dutch questionnaire for assessing breast edema. Moreover, a score cut-off point of 8.5 is determined. This score discriminates between patients who have breast edema and those who have not [34]. In conclusion, the BrEQ is a useful tool to assess and diagnose breast edema in clinical practice and to detect its impact on daily functioning. An English translation of the BrEQ is provided in the Appendix (see Additional file 1).

**Table 1:** Clinimetric properties of the Breast Edema Questionnaire (BrEQ)

Clinimetric property	Breast edema symptoms (part 1)	Activity limitations / participation restrictions (part 2)
Content validity	Good for part 1 and part 2	
Convergent validity	Breast symptoms separately correlated moderately with skin thickness Total symptom score correlated strongly with skin thickness	Total score of activity limitations correlated moderately with - global health status (subscale EORTC QLQ C30) - physical functioning (subscale EORTC QLQ C30) - role functioning (subscale EORTC QLQ C30) - total score of the McGill Quality of Life Questionnaire Total score of activity limitations correlated strongly with - physical wellbeing (subscale McGill QOL questionnaire)
Known-groups validity	Patients with breast edema (diagnosed with US) have a significant higher total symptom score compared to patients without breast edema	Patients with breast edema score significantly higher on activity limitations compared to patients without breast edema
Test-retest reliability	Reliability is strong for the total symptom score Reliability is between strong and moderate for the separate symptoms	Reliability is strong for the total score of activity limitations
Cut-off value	A score cut-off point of ≥8.5 discriminates between patients with breast edema and those without (therefore a score of 9 or higher warrants the diagnosis of breast edema)	/

### Longitudinal course

Several studies investigated the natural course of breast edema over time and demonstrated similar findings [5, 15, 23, 29, 37, 38]. In Table 2 an overview of the available literature in which all assessment methods and all definitions of breast edema are included, is presented. In female breast cancer patients who underwent BCS in combination with radiotherapy, a peak in prevalence was observed after termination of radiotherapy. Afterwards, a gradual spontaneous decline can be expected in the following months [40].

**Table 2:** Time course of breast edema in scientific literature

Reference	Follow-up	Breast edema prevalence
Verbelen (own data, not published)	Prior to RT	52.5%
	After termination of RT	63.8%
	3 months after RT	55.3%
	6 months after RT	57.1%
	12 months after RT	47.5%
Adriaenssens 2012 [5]	0–3 months postoperative	93.3%
	3–6 months postoperative	73.3%
	6–12 months postoperative	82.4%
	12–24 months postoperative	80.6%
	24–60 months postoperative	65.4%
Berrang 2011 [29]	Prior to RT	32%
	1 year after RT	16%
	3 years after RT	6%
Vicini 2007 [15]	> 6 months after RT	32%
	> 24 months after RT	22%
	> 36 months after RT	0%
Young-Afat 2019 [38]	Baseline: prior to RT	12.0%
	3 months after baseline	7.1%
	6 months after baseline	12.4%
	12 months after baseline	8.2%
	18 months after baseline	5.5%
Olivotto 1996 [23]	Prior to RT	26.6%
	3 year after RT	4.3%
	5 years after RT	2.6%
Johansson 2015 [37]	Prior to RT	29%
	2 weeks after RT	39%
	3 months after RT	63%
	6 months after RT	63%
	12 months after RT	39%
	24 months after RT	28%
Lam 2020 [39] (meta-analysis)	0–4 weeks after RT	26.2–47.1%
	6 months – 10 years after RT	7.2–9.9%

Most studies, apart from Adriaenssens et al. are based on the timing of RT to describe the time course of breast edema. Data concerning the amount of time post-operatively is not available

Based upon the findings of Lam 2020 (a meta-analysis); about 7–10% of the patients will need treatment for breast edema provoked by BCS and radiotherapy

*RT* radiation therapy

**Fig. 2:**
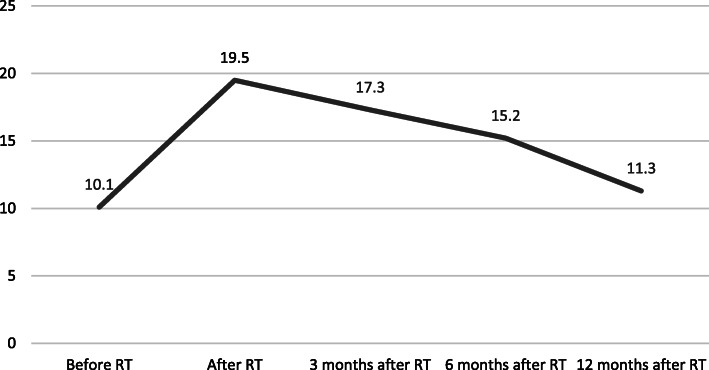
BrEQ-scores on a total score of 80 on different time points

The degree of breast edema has about the same timeline as its prevalence. Figure 2 shows the BrEQ-scores on 80 up until 12 months after radiotherapy. Few studies investigated its degree longitudinally. Wratten et al. described the time course of cutaneous breast edema based on the increase in epidermal thickness, measured with US. In most breast cancer patients who underwent BCS and radiotherapy, epidermal thickness usually peaks at 4 to 6 months post-treatment and in most instances show signs of returning to baseline, 12 months post-treatment. The course of parenchymal breast edema has about the same timeline [11].

In many patients, breast edema is already present prior to radiotherapy. This can be explained by several factors. First, the fact that BCS itself causes breast edema, due to damage to the lymphatic system. This compromises lymphatic transport and could therefore cause breast edema [35]. Second, after BCS, breast edema could be mistaken for typical post-operative complaints such as pain, swelling, tensed skin, etcetera, which aren’t in fact directly associated with breast edema.

A spontaneous decline in breast edema symptoms within 6 months after termination of radiotherapy, can be referred to as transient breast edema. In case the breast symptoms show no signs of return more than 6 months post-radiation, it is called persistent breast edema. We strongly advise patients and health care workers involved in the treatment and after-treatment of breast cancer patients to closely monitor breast complaints after radiotherapy. In cases of mild breast symptoms and/or transient breast edema, treatment is not necessary. Patients with persistent breast edema and/or patients in who the breast complaints are very pronounced and bothersome are recommended to get appropriate treatment.

### Conservative treatment of breast edema

The current evidence based treatment for lymphedema of all sorts is the CDT, which is generally accepted as consensus treatment [41, 42]. However, some aspects of the CDT, namely the manual lymphatic drainage (MLD) are up for debate [43–49]. Although literature on the treatment of breast edema in specific is scarce, we recommend to extrapolate the CDT, which is thoroughly described for the extremities, for breast edema as well, to the utmost extent. CDT is currently the consensus treatment for lymphedema and consists of 4 main pillars: skin care, MLD, compression (bandaging and/or compressions garments) and exercise. The CDT is divided into 2 phases. The goal of phase 1, the intensive phase, is to reduce the swelling. The 4 components of phase 1 are skin care, MLD, compression using bandaging and exercise. Phase 2 aims at preserving the results of phase 1. It contains the same components as in phase 1, except for compression which is generally provided by compression garments instead of bandages. What follows is a synopsis of the 4 pillars of the CDT, and if applicable its evidence for breast edema.

The purpose of *skin care* is to maintain a healthy skin barrier. Damaged and dry skin can become an entry point for infection. Therefore, good skin hygiene, precautionary measures and wound prevention can reduce the risk of infection and possible worsening of the breast edema. Patients are instructed to wash the skin daily with neutral soaps, dry the skin thoroughly with attention for the inframammary fold and to use low pH lotions and emollients. In addition, patients are recommended to take precautionary measures. Besides skin hygiene, recommendations supported by scientific evidence for lymphedema in general are as follows: avoid trauma, disinfect and treat wounds immediately, avoid sauna visits and seek medical help in case of skin changes [42]. Additional information given to the patients can be relevant as well since they were proven to be risk factors for aggravating lymphedema. Therefore, these recommendations rely on common sense: maintain or achieve a healthy/normal BMI, protect the skin from sunburn and wear appropriate clothing and bra [42]. For breast edema in specific, risk factors are investigated in a systematic review of the literature [35]. Table 3 gives an overview of the risk factors found in literature, however consensus among studies is lacking. Also, those risk factors are not likely to be reversible by actions of the patients.

**Table 3:** Risk factors for breast edema

Related to radiotherapy	Increase in irradiated breast volume
	Increase in boost volume
	Photon boost
	Increasing breast separation
	External beam radiation (vs. intra-operative radiotherapy)
	Conventional radiotherapy (vs. intensity-modulated radiotherapy)
Related to surgery	Postoperative infection
Related to tumor characteristics	Larger tumor
Related to personal factors	Larger breast volume
	Increasing breast density
	Diabetes mellitus

*MLD* is another pillar of the CDT which can be performed both in the intensive and maintenance phase. MLD is a massage technique that aims to promote the movement of lymphatic fluid out of the swollen area as well as the uptake of interstitial fluid by the lymphatic system [50]. Although MLD is a well-established treatment modality for lymphedema of the extremities in clinical practice, its effectiveness is still questioned among researchers [43–49]. For breast edema, scientific research concerning MLD is missing, although it is often administered in clinical practice. Lymph fluid from the breast is drained proximally towards the axillary and supraclavicular lymph nodes and/or towards the lymph nodes of the contralateral side. Evidence needs to be established in order to determine whether MLD should be omitted definitively from the CDT for breast edema or not. Nevertheless, currently, awaiting evidence concerning the role of MLD, it is our recommendation to exclude MLD from the breast edema treatment, as it is time consuming en costly.

**Fig. 3:**
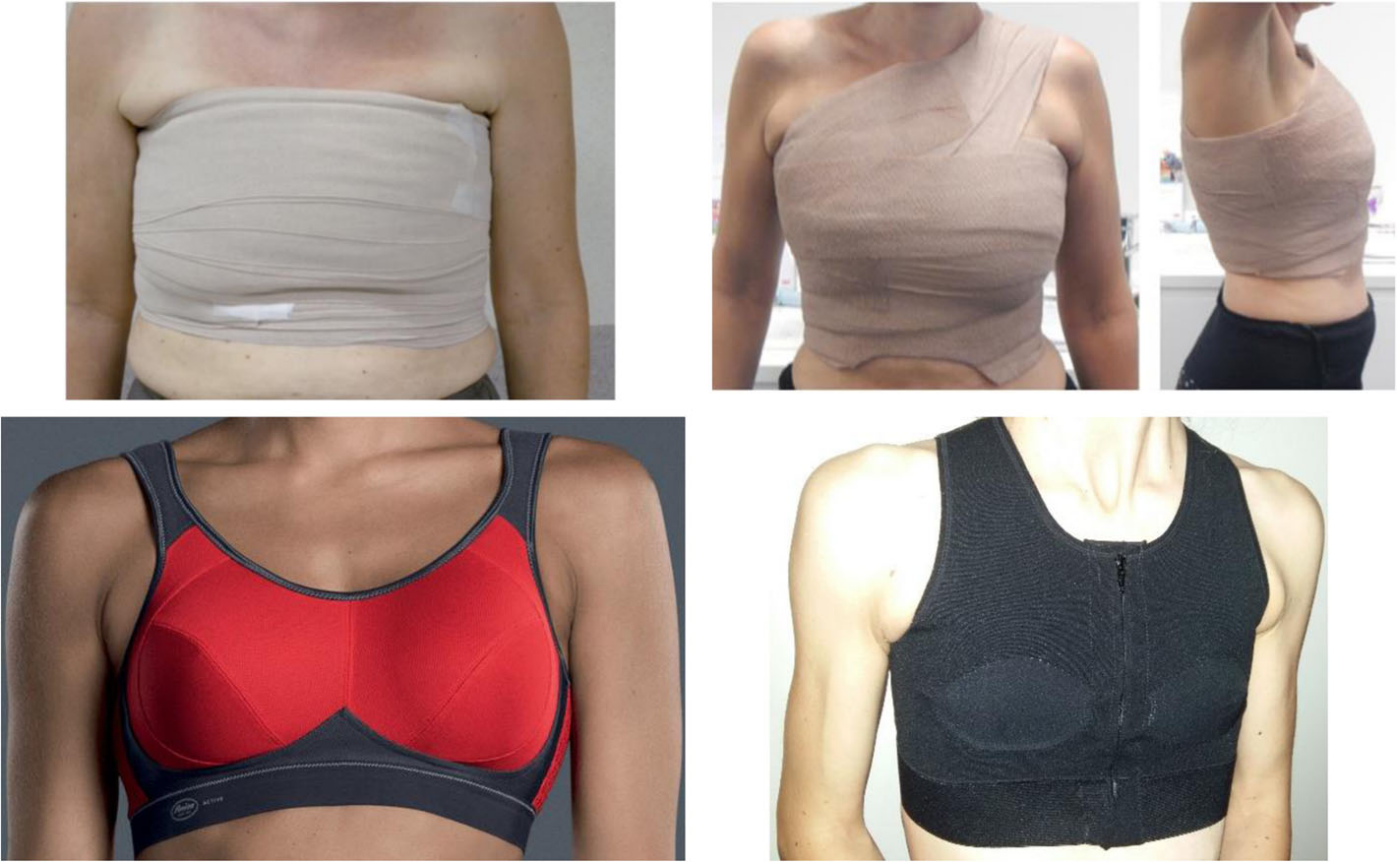
Overview of compression therapy for breast edema. During edema reduction therapy short stretch bandages as well as 2-layer self-adhesive compression systems can be used. During the maintenance phase, a sports bra or custom made compression bra can be used. The sports bra is sometimes used as preventative therapy as well, currently strong evidence of the preventative effect is lacking

During the intensive phase of the CDT, *compression* (see pictures, Fig. 3) is used in order to decrease the lymphedema volume for which most commonly, short-stretch multilayer bandages are used [50]. However, for breast edema it is difficult to apply the bandages correctly and with appropriate pressure and many women find it uncomfortable to wear. Therefore, a compression bra or sports bra of compression type can be provided instead. During the maintenance phase, the use of this type of bra can be continued. Importantly, scientific evidence concerning compression therapy for women with breast edema is scarce. A study of Johansson et al. investigated the treatment of breast edema using a sports bra of compression type with firm pressure flattening the breasts and compared it with ordinary bras [32]. This type of compression needed to be worn during daytime for 9 months. Results showed that this breast compression treatment had no effect on symptoms of breast edema and on the amount of local tissue water measured by the TDC. Therefore, the recommendation is to wear a sports bra of compression type, only if it doesn’t cause a negative impact on comfort. Additionally, closely monitor the symptoms of breast edema in order to intervene if necessary. It is needless to say that more research concerning this topic is of great importance.

It has consistently been demonstrated that *exercise* is beneficial for managing lymphedema, as well aerobic exercise as resistance training. However, only 1 study investigated whether women with breast edema would respond similarly to exercise than to those with arm edema [51]. This study investigated a supervised 12-week combined aerobic and resistance training program. The exercise group reported a greater reduction in breast-related symptoms than the control group, assessed by the EORTC BR23 breast symptom questions. Measures of extracellular fluid, assessed with bioimpedance spectroscopy ratio, decreased in the exercise group compared to the control group. No significant difference was detected in dermal thickness in the breast, assessed by ultrasound [51]. Improving the use of a muscle pump will stimulate the lymphatic transport and improving the overall physical endurance and strength will lead to a better physical condition and coping [42]. Importantly, strenuous exercise will not aggravate the lymphedema which is often falsely assumed [51, 52]. Therefore, they should not be avoided unless they provoke pain or articular problems.

### Follow-up assessments

During the follow-up of a patient treated for breast edema, several assessments can be performed to determine treatment results. First, the BrEQ can be used. If during the treatment the BrEQ-score decreases to a value below the cut-off point of 8.5 this signifies a good result. Additionally, part 2 of the BrEQ can be used to monitor the impact of breast edema on quality of life and activities of daily living [34]. Of course a clinical examination can be performed periodically, especially to determine whether or not the pitting sign has disappeared completely. If pitting is absent, breast edema has been reduced. A more technical assessment that can be performed is the assessment of TDC. TDC ratios have been demonstrated as prognostic in the presence of edema [32, 53, 54]. In patients with bilateral edema, no TDC ratios can be calculated. For these patients the progression in TDC- value (a percentage of water) can be monitored.

## Clinical implications

Breast edema can be a serious complaint which cannot be neglected. Etiologies for breast edema are versatile, which makes an accurate diagnosis of the underlying condition important. In case of breast edema after BCS and radiotherapy, it is recommended that all patients who receive this type of breast cancer treatment at least get informed about this forgotten complaint. In case of breast edema of another etiology, it is mandatory to rule out malignancies or other treatable causes.

In addition, similarities between breast edema and radiodermatitis can be observed, like for example edema, redness, hardness and pain [55]. It is not always possible to distinguish between both conditions. Breast edema, however, can be present prior to radiotherapy. We advise patients and health care workers to monitor breast complaints closely, and to intervene if necessary. To aid in the detection and monitoring of breast edema, we suggest to use the BrEQ in combination with a physical examination. This method is fast and doesn’t require much material or resources.

Breast edema follows a natural course in which we see a spontaneous decline in the months after radiotherapy. Furthermore, breast edema is often subclinical and therefore not recognized and acknowledged by health care workers, because breast complaints are mild. For those reasons, not all patients need treatment for breast edema. The take home message should be to closely monitor those patients in who the BrEQ-score doesn’t decline within 6 months after termination of radiotherapy and provide them with the appropriate therapy. We recommend a morbidity screening after breast cancer treatment on regular basis. Self-assessment using a checklist or smartphone application are both feasible approaches.

Since evidence concerning the treatment of breast edema is currently sparse, we recommend the CDT, by analogy with the lymphedema treatment of the extremities. However, we recommend omitting MLD, since its evidence is low. Therefore, the breast edema treatment involves skin care, exercise therapy and compression. Additionally, a patients should be informed about the normal course of breast edema development.

**Table:** 

**Take home messages:**	
- **Patients should be informed about breast edema and its natural course.**	
- **Patients treated with BCS and radiotherapy should be monitored till 12 months after the end of radiotherapy**	
- **To aid in the detection and monitoring of breast edema, the use of the BrEQ in combination with a physical examination is a suitable approach.**	
- **If no spontaneous decline of breast edema after 6 months is seen and no other treatable cause is found; start treating the edema**	
- **Currently, CDT, with the exception of MLD, is the recommended treatment which involves skin care, compression and exercise therapy. However, strong scientific evidence still needs to be established.**	

## Future research priorities

Long term prospective research is vital to gain better insight in breast edema as a morbidity after BCS and radiotherapy. Especially, since some patients still suffer from breast edema years after surgery. A longitudinal study could make it possible to detect when problems arise and could therefore be valuable to determine when appropriate treatment or sufficient information should be provided.

An international consensus should be reached among clinicians and researchers concerning the definition of breast edema. Furthermore, we need to consider a standardized assessment tool which could serve as a gold standard. The BrEQ could be considered as a gold standard since it covers all the domains of disability according to the ICF framework (www.who.int/classifications/icf/en). This Dutch questionnaire is the first to specifically assess breast edema. A translation (currently a Spanish, Turkish and English version are being prepared) and a further investigation of the degree to which the items on a translated BrEQ adequately reflect the items on the original Dutch version, is mandatory. Moreover, it is important to encourage researchers to consistently report whenever a modified version of the BrEQ is used.

Concerning the treatment of breast edema, high quality studies are necessary to prove the effectiveness of the CDT for breast edema in specific. Furthermore, the appropriate timing and specific content of the treatment program need to be further investigated. There could be a rationale for other treatment modalities like for example fascia release techniques, however, evidence for breast edema is currently lacking. Additionally, more attention and more scientific research should go to the treatment of skin complaints (including scar tissue treatment if necessary) and the importance of compression and exercise therapy.

## Conclusion

Breast edema is a common complaint after BCS and radiotherapy, however little described in scientific literature. Sufficient information concerning the diagnosis, longitudinal course and treatment of breast edema should reach health care workers involved in breast cancer treatment in order to improve care for these patients.

## Supplementary Information

**Additional file 1.** Breast edema questionnaire (BrEQ) – English version. Note: The English translation of the BreQ has not yet been validated.

## Data Availability

The datasets used and/or analysed during the current study are available from the corresponding author on reasonable request.

## Abbreviations

BCS: Breast conserving surgery
BrEQ: Breast edema questionnaire
CDT: Complex decongestive therapy
ICF: International Classification of Functioning, Disability and Health
MLD: Manual lymphatic drainage
TDC: Tissue dielectric constant

## References

[1] Ferlay J, Steliarova-foucher E, Lortet-tieulent J, Rosso S (2013). Cancer incidence and mortality patterns in Europe : estimates for 40 countries in 2012. Eur J Cancer.

[2] Thomas DB, Moe RE, White E. Breast Conservation Therapy in the United States following the 1990 National Institutes of Health consensus development conference on the treatment of patients with early stage invasive. Published online 1999:628–637. 10440690

[3] Kwak JY, Kim EK, Chung SY (2005). Unilateral breast edema: Spectrum of etiologies and imaging appearances. Yonsei Med J.

[4] Harsolia A, Kestin L, Grills I (2007). Intensity-modulated radiotherapy results in significant decrease in clinical toxicities compared with conventional wedge-based breast radiotherapy. Int J Radiat Oncol Biol Phys.

[5] Adriaenssens N, Verbelen H, Lievens P, Lamote J (2012). Lymphedema of the operated and irradiated breast in breast cancer patients following breast conserving surgery and radiotherapy. Lymphology..

[6] Adriaenssens N, Belsack D, Buyl R (2012). Ultrasound elastography as an objective diagnostic measurement tool for lymphoedema of the treated breast in breast cancer patients following breast conserving surgery and radiotherapy. Radiol Oncol.

[7] Toledano A, Garaud P, Serin D (2006). Concurrent administration of adjuvant chemotherapy and radiotherapy after breast-conserving surgery enhances late toxicities: long-term results of the ARCOSEIN multicenter randomized study. Int J Radiat Oncol Biol Phys.

[8] Clarke D, Martinez A, Cox RS, Goffinet DR (1982). Breast edema following staging axillary node dissection in patients with breast carcinoma treated by radical radiotherapy. Cancer..

[9] Delay E, Gosset J, Toussoun G, Delaporte T, Delbaere M (2008). Post-treatment sequelae after breast cancer conservative surgery. Ann Chir Plast Esthet.

[10] Pezner RD, Patterson MP, Hill LR, Desai KR, Vora N, Lipsett JA (1985). Breast edema in patients treated conservatively for stage I and II breast cancer. Int J Radiat Oncol Biol Phys.

[11] Wratten CR, O’brien PC, Hamilton CS, Bill D, Kilmurray J, Denham JW (2007). Breast edema in patients undergoing breast-conserving treatment for breast cancer: assessment via high frequency ultrasound. Breast J.

[12] Poglio S, Galvani S, Bour S, André M, Prunet-Marcassus B, Pénicaud L (2009). Adipose tissue sensitivity to radiation exposure. Am J Pathol.

[13] Constantine C, Parhar P, Lymberis S (2008). Feasibility of accelerated whole-breast radiation in the treatment of patients with ductal carcinoma in situ of the breast. Clin Breast Cancer.

[14] Wenz F, Welzel G, Keller A (2008). Early initiation of external beam radiotherapy (EBRT) may increase the risk of long-term toxicity in patients undergoing intraoperative radiotherapy (IORT) as a boost for breast cancer. Breast.

[15] Vicini FA, Chen P, Wallace M (2007). Interim cosmetic results and toxicity using 3D conformal external beam radiotherapy to deliver accelerated partial breast irradiation in patients with early-stage breast cancer treated with breast-conserving therapy. Int J Radiat Oncol Biol Phys.

[16] Mussari S, Sabino Della Sala W, Busana L (2006). Full-dose intraoperative radiotherapy with electrons in breast cancer. First report on late toxicity and cosmetic results from a single-institution experience. Strahlenther Onkol.

[17] Marcenaro M, Sacco S, Pentimalli S (2004). Measures of late effects in conservative treatment of breast cancer with standard or hypofractionated radiotherapy. Tumori..

[18] Back M, Guerrieri M, Wratten C, Steigler A (2004). Impact of radiation therapy on acute toxicity in breast conservation therapy for early breast Cancer. Clin Oncol.

[19] Hoeller U, Tribius S, Kuhlmey A, Grader K, Fehlauer F, Alberti W (2003). Increasing the rate of late toxicity by changing the score? A comparison of RTOG/EORTC and LENT/SOMA scores. Int J Radiat Oncol Biol Phys.

[20] Grann A, McCormick B, Chabner ES (2000). Prone breast radiotherapy in early-stage breast cancer: a preliminary analysis. Int J Radiat Oncol.

[21] Kuptsova N, Chang-Claude J, Kropp S (2008). Genetic predictors of long-term toxicities after radiation therapy for breast cancer. Int J Cancer.

[22] Goyal S, Daroui P, Khan AJ, Kearney T, Kirstein L, Haffty BG (2013). Three-year outcomes of a once daily fractionation scheme for accelerated partial breast irradiation (APBI) using 3-D conformal radiotherapy (3D-CRT). Cancer Med.

[23] Olivotto IA, Weir LM, Kim-Sing C (1996). Late cosmetic results of short fractionation for breast conservation. Radiother Oncol.

[24] Dragun AE, Quillo AR, Riley EC (2013). A phase 2 trial of once-weekly Hypofractionated breast irradiation: first report of acute toxicity, feasibility, and patient satisfaction. Int J Radiat Oncol.

[25] Chadha M, Vongtama D, Friedmann P (2012). Comparative acute toxicity from whole breast irradiation using 3-week accelerated schedule with concomitant boost and the 6.5-week conventional schedule with sequential boost for early-stage breast Cancer. Clin Breast Cancer.

[26] Kelemen G, Varga Z, Lázár G, Thurzó L, Kahán Z (2012). Cosmetic outcome 1-5 years after breast conservative surgery, irradiation and systemic therapy. Pathol Oncol Res.

[27] Li F, He Z, Xue M, Chen L, Wu S, Guan X, Li F, He Z, Xue M, Chen L, Wu S, Guan X (2011). Feasibility and acute toxicity of 3-dimensional conformal external-beam accelerated partial-breast irradiation for early-stage breast cancer after breast-conserving surgery in Chinese female patients. Chin Med J Chin Med J (Engl).

[28] Barnett GC, Wilkinson JS, Moody AM (2011). The Cambridge breast intensity-modulated radiotherapy trial: patient- and treatment-related factors that influence late toxicity. Clin Oncol.

[29] Berrang TS, Olivotto I, Kim D-H (2011). Three-year outcomes of a Canadian multicenter study of accelerated partial breast irradiation using conformal radiation therapy. Int J Radiat Oncol Biol Phys.

[30] Kuzmiak CM, Zeng D, Cole E, Pisano ED (2009). Mammographic findings of partial breast irradiation. Acad Radiol.

[31] Forrai G, Polgar C, Zana K (2001). The role of STIR MRI sequence in the evaluation of the breast following conservative surgery and radiotherapy. Neoplasma..

[32] Johansson K, Jönsson C, Björk-Eriksson T (2020). Compression treatment of breast edema: a randomized controlled pilot study. Lymphat Res Biol.

[33] Formenti SC, Hsu H, Fenton-Kerimian M (2012). Prone accelerated partial breast irradiation after breast-conserving surgery: five-year results of 100 patients. Int J Radiat Oncol.

[34] Verbelen H, Vrieze T De, Soom T Van, Meirte J, Goethem M Van, Hufkens G. Development and clinimetric properties of the Dutch Breast Edema Questionnaire ( BrEQ ‑ Dutch version ) to diagnose the presence of breast edema in breast cancer patients. Qual Life Res. 2020;29(2):569-78. 10.1007/s11136-019-02337-z 31659592

[35] Verbelen H, Gebruers N, Beyers T, De Monie A-C, Tjalma W. Breast edema in breast cancer patients following breast-conserving surgery and radiotherapy: a systematic review. Breast Cancer Res Treat. 2014;147(3):463-71. 10.1007/s10549-014-3110-8 25164973

[36] Wratten C, Kilmurray J, Wright S, Back M, Hamilton CS, Denham JW (2000). Pilot study of high-frequency ultrasound to assess cutaneous Oedema in the conservatively managed breast. Radiat Oncol Invest.

[37] Johansson K, Darkeh MH, Lahtinen T, Björk-Eriksson T, Alexsson R (2015). Two-year follow-up of temporal changes of breast edema after breast cancer treatment with surgery and radiation evaluated by tissue dielectric constant (TDC). Eur J of Lymphol.

[38] Young-Afat DA, Gregorowitsch ML, van den Bongard DH (2019). Breast edema following breast-conserving surgery and radiotherapy: patient-reported prevalence, determinants, and effect on health-related quality of life. JNCI Cancer Spectr.

[39] Lam E, Yee C, Wong G (2020). A systematic review and meta-analysis of clinician-reported versus patient-reported outcomes of radiation dermatitis. Breast..

[40] Verbelen H (2020). Arm, shoulder and breast morbidity after breast cancer treatment, PhD dissertation, University of Antwerp.

[41] Society TI, Document C, Congress XVII (2016). The diagnosis and treatment of peripheral lymphedema: 2016 consensus document of the international society of lymphology. Lymphology..

[42] Gebruers N, Verbelen H, De Vrieze T, et al. Current and future perspectives on the evaluation, prevention and conservative management of breast cancer related lymphoedema: a best practice guideline. Eur J Obstet Gynecol Reprod Biol. 2017;216. 10.1016/j.ejogrb.2017.07.035 28811052

[43] Thompson B, Gaitatzis K, Janse de Jonge X, Blackwell R, Koelmeyer LA. Manual lymphatic drainage treatment for lymphedema: a systematic review of the literature. J Cancer Surviv. 2020. 10.1007/s11764-020-00928-1. [Epub ahead of print]. 10.1007/s11764-020-00928-1 32803533

[44] Stuiver MM, ten Tusscher MR, Agasi-Idenburg CS, Lucas C, Aaronson NK, Bossuyt PMM. Conservative interventions for preventing clinically detectable upper-limb lymphoedema in patients who are at risk of developing lymphoedema after breast cancer therapy. Cochrane Database Syst Rev. 2015;2. 10.1002/14651858.CD009765.pub2 PMC10651942 25677413

[45] Ezzo J, Manheimer E, Mcneely ML, Howell DM, Weiss R, Johansson KI, et al. Manual lymphatic drainage for lymphedema following breast cancer treatment. Cochrane Database Syst Rev. 2015;5. 10.1002/14651858.CD003475.pub2 PMC4966288 25994425

[46] Huang TW, Tseng SH, Lin CC, Bai CH, Chen CS, Hung CS, et al. Effects of manual lymphatic drainage on breast cancer-related lymphedema: A systematic review and meta-analysis of randomized controlled trials. World J Surg Oncol [journal on the internet]. 2013;11:15. 10.1186/1477-7819-11-15. 10.1186/1477-7819-11-15 PMC3562193 23347817

[47] Tambour M, Holt M, Speyer A, Christensen R, Gram B (2018). Manual lymphatic drainage adds no further volume reduction to complete decongestive therapy on breast cancer-related lymphoedema: a multicentre, randomised, single-blind trial. Br J Cancer.

[48] Gradalski T, Ochalek K, Kurpiewska J (2015). Complex decongestive lymphatic therapy with or without Vodder II manual lymph drainage in more severe chronic Postmastectomy upper limb lymphedema: a randomized noninferiority prospective study. J Pain Symptom Manag.

[49] Andersen L, Hojris I, Erlandsen M, Andersen J (2000). Treatment of breast-cancer-related lymphedema with or without manual lymphatic drainage: a randomized study. Acta Oncol.

[50] Executive Committee of the International Society of Lymphology. The diagnosis and treatment of peripheral lymphedema: 2020 Consensus Document of the International Society of Lymphology. Lymphology. 2020;53(1):3-19. 32521126

[51] Kilbreath SL, Ward LC, Davis GM, et al. Reduction of breast lymphoedema secondary to breast cancer: a randomised controlled exercise trial. Breast Cancer Res Treat. 2020;184(2):459-67. 10.1007/s10549-020-05863-4 32812177

[52] Bloomquist K, Oturai P, Steele ML (2018). Heavy-load lifting: acute response in breast cancer survivors at risk for lymphedema. Med Sci Sports Exerc.

[53] Mayrovitz HN, Weingrad HN, Brlit F, Lopez LB, Desfor R (2015). Tissue dielectric constant (TDC) as an index of localized arm skin water: differences between measuring probes and genders. Lymphology..

[54] Koehler LA, Mayrovitz HN (2020). Tissue dielectric constant measures in women with and without clinical trunk lymphedema following breast Cancer surgery: a 78-week longitudinal study. Phys Ther.

[55] Hegedus F, Mathew LM, Schwartz RA (2017). Radiation dermatitis: an overview. Int J Dermatol.

