# Supplementary material for: Breast edema, from diagnosis to treatment: state of the art
Source: Arch Physiother. 2021 Mar 29;11:8. doi: 10.1186/s40945-021-00103-4 (PMC8006345; doi:10.1186/s40945-021-00103-4)
Supplement: Supplementary file 1 — Additional file 1. Breast edema questionnaire (BrEQ) – English version. Note: The English translation of the BreQ has not yet been validated. [file 40945_2021_103_MOESM1_ESM.docx]

**Breast Edema Questionnaire**

**Instructions**

This questionnaire aims to gain information about your complaints. Please indicate the extent to which you have experienced complaints at this moment.

Completing this questionnaire only takes a few minutes of your time.

- When mentioned ‘(Circle)’ we ask you to circle the answer that applies to you.
- When mentioned ‘(Fill in)’, we ask you for a short and concise description.

**Thank you in advance for your cooperation and your valuable time**

**Name: ____________________________________________**

| **BREAST COMPLAINTS** |
| --- |

| **1.** | Indicate to what extent you suffered from following complaints in the operated breast during the past week. (Circle)   \|  \| **Not at**  **all** \| \| \|  \|  \|  \|  \|  \| **Very severe** \| \| \| \| --- \| --- \| --- \| --- \| --- \| --- \| --- \| --- \| --- \| --- \| --- \| --- \| \| **Pain** in the operated breast \| 0 \| 1 \| 2 \| 3 \| 4 \| 5 \| 6 \| 7 \| 8 \| 9 \| 10 \| \| A feeling of **heaviness** in the operated breast \| 0 \| 1 \| 2 \| 3 \| 4 \| 5 \| 6 \| 7 \| 8 \| 9 \| 10 \| \| A **swollen** breast at the operated side \| 0 \| 1 \| 2 \| 3 \| 4 \| 5 \| 6 \| 7 \| 8 \| 9 \| 10 \| \| The **skin** feels **tensed** at the operated breast \| 0 \| 1 \| 2 \| 3 \| 4 \| 5 \| 6 \| 7 \| 8 \| 9 \| 10 \| \| **Redness** of the skin at the operated breast \| 0 \| 1 \| 2 \| 3 \| 4 \| 5 \| 6 \| 7 \| 8 \| 9 \| 10 \| \| A **print** of my bra is visible at the operated breast \| 0 \| 1 \| 2 \| 3 \| 4 \| 5 \| 6 \| 7 \| 8 \| 9 \| 10 \| \| The **pores** of the skin at the operated breast are **enlarged** \| 0 \| 1 \| 2 \| 3 \| 4 \| 5 \| 6 \| 7 \| 8 \| 9 \| 10 \| \| The operated breast feels **hard** at some places \| 0 \| 1 \| 2 \| 3 \| 4 \| 5 \| 6 \| 7 \| 8 \| 9 \| 10 \|   **Since your surgery, have you had any other complaints at the operated breast during the past week? If yes, please describe.** (Fill in) |
| --- | --- | --- | --- | --- | --- | --- | --- | --- | --- | --- | --- | --- | --- | --- | --- | --- | --- | --- | --- | --- | --- | --- | --- | --- | --- | --- | --- | --- | --- | --- | --- | --- | --- | --- | --- | --- | --- | --- | --- | --- | --- | --- | --- | --- | --- | --- | --- | --- | --- | --- | --- | --- | --- | --- | --- | --- | --- | --- | --- | --- | --- | --- | --- | --- | --- | --- | --- | --- | --- | --- | --- | --- | --- | --- | --- | --- | --- | --- | --- | --- | --- | --- | --- | --- | --- | --- | --- | --- | --- | --- | --- | --- | --- | --- | --- | --- | --- | --- | --- | --- | --- | --- | --- | --- | --- | --- | --- | --- | --- |
| **2.** | **Indicate** (circle) **on a scale from 0 to 10 if you had difficulty with following activities as a result of the breast complaints during the past week. PLEASE NOTE: we only assess activity limitations due to breast complaints and not due to arm complaints. If the activity does not apply to you, please indicate it in the column N/A”.**   \|  \| **No Unbearable**  **complaints complaints** \| **N/A** \| \| --- \| --- \| --- \| \| Sleeping \| 0 1 2 3 4 5 6 7 8 9 10 \|  \| \| Lying down \| 0 1 2 3 4 5 6 7 8 9 10 \|  \| \| Sitting \| 0 1 2 3 4 5 6 7 8 9 10 \|  \| \| Standing \| 0 1 2 3 4 5 6 7 8 9 10 \|  \| \| Vocational activities \| 0 1 2 3 4 5 6 7 8 9 10 \|  \| \| Household chores \| 0 1 2 3 4 5 6 7 8 9 10 \|  \| \| Driving a car \| 0 1 2 3 4 5 6 7 8 9 10 \|  \| \| Handicraft \| 0 1 2 3 4 5 6 7 8 9 10 \|  \| \| Walking \| 0 1 2 3 4 5 6 7 8 9 10 \|  \| \| Sports \| 0 1 2 3 4 5 6 7 8 9 10 \|  \| \| Getting (un)dressed \| 0 1 2 3 4 5 6 7 8 9 10 \|  \| \| Putting on a bra \| 0 1 2 3 4 5 6 7 8 9 10 \|  \| \| Wearing a bra \| 0 1 2 3 4 5 6 7 8 9 10 \|  \| \| Computer work \| 0 1 2 3 4 5 6 7 8 9 10 \|  \| \| Other activities (Fill in)  _____________________  _____________________ \| 0 1 2 3 4 5 6 7 8 9 10  0 1 2 3 4 5 6 7 8 9 10 \|  \| |
| **3.** | **Wich of the above mentioned activity limitations disturbs you the most in your daily living? Please give your top 3.** (Fill in)   1. **____________________________________________** 2. **____________________________________________** 3. **____________________________________________** |
